# *HvDep1* Is a Positive Regulator of Culm Elongation and Grain Size in Barley and Impacts Yield in an Environment-Dependent Manner

**DOI:** 10.1371/journal.pone.0168924

**Published:** 2016-12-22

**Authors:** Toni Wendt, Inger Holme, Christoph Dockter, Aileen Preuß, William Thomas, Arnis Druka, Robbie Waugh, Mats Hansson, Ilka Braumann

**Affiliations:** 1 Carlsberg Research Laboratory, Copenhagen, Denmark; 2 Department of Molecular Biology and Genetics, Faculty of Science and Technology, Aarhus University, Slagelse, Denmark; 3 James Hutton Institute, Invergowrie, Dundee, United Kingdom; 4 Division of Plant Sciences, The University of Dundee, Invergowrie, Dundee, United Kingdom; 5 Department of Biology, Lund University, Lund, Sweden; National Taiwan University, TAIWAN

## Abstract

Heterotrimeric G proteins are intracellular membrane-attached signal transducers involved in various cellular processes in both plants and animals. They consist of three subunits denoted as α, β and γ. The γ-subunits of the so-called AGG3 type, which comprise a transmembrane domain, are exclusively found in plants. In model species, these proteins have been shown to participate in the control of plant height, branching and seed size and could therefore impact the harvestable yield of various crop plants. Whether AGG3-type γ-subunits influence yield in temperate cereals like barley and wheat remains unknown. Using a transgenic complementation approach, we show here that the Scottish malting barley cultivar (cv.) Golden Promise carries a loss-of-function mutation in *HvDep1*, an AGG3-type subunit encoding gene that positively regulates culm elongation and seed size in barley. Somewhat intriguingly, agronomic field data collected over a 12-year period reveals that the *HvDep1* loss-of-function mutation in cv. Golden Promise has the potential to confer either a significant increase or decrease in harvestable yield depending on the environment. Our results confirm the role of AGG3-type subunit-encoding genes in shaping plant architecture, but interestingly also indicate that the impact *HvDep1* has on yield in barley is both genotypically and environmentally sensitive. This may explain why widespread exploitation of variation in AGG3-type subunit-encoding genes has not occurred in temperate cereals while in rice the *DEP1* locus is widely exploited to improve harvestable yield.

## Introduction

Barley (*Hordeum vulgare* L.) was one of the first crops domesticated in the Fertile Crescent about 10,000 years ago [1]. It remains important in modern agriculture, ranking fifth after maize, wheat, rice, and soybean in worldwide dry matter production [2]. Barley grains are widely used as animal feed and are the major raw material for the malting and brewing industries. In several geographical regions, barley is used for human consumption, and due to its health benefits in human diets, the interest of using barley as food is increasing, especially in the western world [3].

Cereal breeding programs face critical challenges for the development of new, future-proof cultivars. A rapidly growing human population, combined with decreasing areas suitable for agriculture and global and local changes in the climate [4] that put agricultural production under pressure, emphasize the need for significant yield increases for all major crops during the next decades.

In cereal crops, plant yield depends on the number, size and weight of the grains per unit area. Genes involved in the regulation of grain size and number, either by variation in the numbers per spike or numbers of fertile spikes, are therefore of particular interest for cereal breeding [5]. The present study was designed to explore the role of genes encoding the plant specific heterotrimeric G protein γ-subunits in the control of both grain yield and plant architecture, as recent studies in Arabidopsis [6, 7] and rice [8, 9] reveal an involvement in determining overall plant morphology, seed size, shape and yield.

Heterotrimeric G proteins are signal transducers found throughout the animal and the plant kingdoms. In plants, heterotrimeric G proteins are known to control diverse functions, such as growth, cell proliferation, defense systems, and hormonal responses [10]. The functional complex consists of separate α-, β- and γ-subunits. The α-subunit is the actual G protein that binds GDP or GTP. A specific feature of plant α-subunits is that they are self-activated by spontaneous release of GDP followed by binding of GTP. In the GTP-bound state, the α-subunit dissociates from the complex, which allows signal transduction via both the α-subunit and the β/γ-complex [10].

In contrast to animals, the repertoire of heterotrimeric G-protein subunits in plants is rather simple [11]. In Arabidopsis only one α-, one β- and three γ-subunits have been identified [12], suggesting, that the function of the heterotrimer differs depending on the respective γ-subunit involved [13]. While the two canonical Arabidopsis heterotrimeric G protein γ-subunits AGG1 and AGG2 were identified at the turn of the millennium [14, 15], the third γ-subunit, AGG3, was first identified in 2011 [6] due to its unusual structural characteristics. AGG3 contains a NH_2_-terminal γ-domain with homology to AGG1 and AGG2, but the molecular mass of AGG3 is twice that of AGG1 and AGG2 due to the presence of a COOH-terminal cysteine-rich domain. This unique type of γ-subunit had not been described before the identification of AGG3 and it seems to be specific for plants [6]. The AGG3-specific COOH-terminal domain was predicted to contain a transmembrane domain followed by a tumor necrosis factor receptor/nerve growth factor receptor (TNFR/NGFR) family type of cysteine-rich signature, as well as four von Willebrand factor type C (VWFC) modules [7]. The initially suggested and recently experimentally proven presence [16] of a transmembrane domain and an extracellular cysteine-rich domain in the COOH-terminus of AGG3 led to the speculation that γ-subunits of the AGG3 type could function as receptors [17].

The AGG3-like γ-subunits are of particular interest in plant breeding as these proteins have been shown to regulate seed and organ size [7]. Li et al. [7] showed that *agg3* loss of function mutants in Arabidopsis develop smaller seeds and organs, while *AGG3* overexpression results in larger seeds and increased organ size [7]. In the monocotyledonous crop plant rice, two AGG3-type γ-subunits are known, namely *Grain size 3* (*GS3*) and *Dense and erect panicle 1* (*DEP1*). The respective genes were described as important quantitative trait loci (QTL) for grain size and yield. Due to the late discovery of AGG3, both GS3 and DEP1 were only recently identified as γ-subunits of the heterotrimeric G-protein [6]. In the case of *GS3* a number of corresponding alleles with different impacts on seed size are known [17]. *GS3-1* and *GS3-2* encode full-length AGG3-type γ-subunit proteins (231 and 232 amino-acid residues, respectively) and are considered wild type alleles. The *GS3-3* allele is characterized by a premature stop codon that results in translation of a 55 amino-acid long protein lacking the γ-domain, the putative transmembrane domain and the COOH-terminal cysteine-rich domain. *GS3-4* encodes a protein of 149 residues with intact γ- and putative transmembrane-domains, but with a partial deletion of the sequence encoding the cysteine-rich domain [9]. Rice plants carrying the *GS3-3* allele develop longer and heavier grains, which increase the thousand grain weight (TGW) by 8 to 27% compared with plants carrying a wild type *GS3* allele. In contrast, rice plants with the *GS3-4* allele develop shorter grains. It should be noted that rice GS3 negatively regulates grain size, while the Arabidopsis AGG3 is a positive regulator of seed size [7].

The second AGG3-type γ-subunit in rice is encoded by *DEP1*, identified as the causative gene for a major grain yield QTL [8]. Plants carrying the mutant allele *dep1* are characterized by a dense and erect panicle, combined with a semi-dwarf stature [8], indicating that AGG3-type γ-subunit encoding genes have a major impact on overall plant architecture. The *dep1* mutation in rice results in a truncated protein of 195 amino-acid residues, that lacks 230 residues at the COOH-terminal end [8]. Structurally, this mutant allele resembles rice *GS3-4*, because it contains the sequence encoding the γ- and the putative transmembrane domains but lacks the COOH-terminal cysteine-rich domain [17]. Rice plants carrying the *dep1* allele develop more grains per panicle, which translates into a grain-yield increase per plant [8]. In contrast, a very similar mutation at the *DEP1* locus, *qPE9-1* confers synthesis of a protein that is characterized by a loss of 231 amino-acid residues from the COOH-terminus. The mutation was shown to result in a decrease in grain yield per plant [18]. The *qPE9-1* allele still positively influences the yield per area. This could be a result of a general improvement of plant architecture, namely the associated semi-dwarfism and the short, erect panicles and leaves [18].

The fact that two AGG3-type subunits are present in rice support the hypothesis that γ-subunits in plants can provide functional selectivity to the heterotrimeric G-protein signaling complex. Manipulating the function of the γ-subunits, especially of the AGG3-type, could therefore alter plant architecture, seed size and grain yield in other major cereal crops such as barley and wheat.

Until today the impact of γ-subunits of the AGG3-type on plant architecture and seed size has not been examined in barley and, unlike the situation in rice, no severe mutations in a barley *AGG3* orthologue are known. However, a recent report documented the presence of 37 SNPs and indels in the genomic sequence of the barley orthologue of the *DEP1* gene in a collection of 167 Canadian, European and African cultivars. None of the nucleotide polymorphisms changed the polypeptide sequence of the HvDEP1 protein [19].

Here we used a near-isogenic line (NIL) collection [20] of morphological barley mutants to identify a barley mutant affected in the *HvDep1* gene. We show, involving a transgenic complementation approach, that the *HvDep1* gene is identical to the barley semi-dwarfing locus *Breviaristatum-e* (*Ari-e*). An *ari-e* mutant allele, *ari-e*.GP, is present in Golden Promise, a successful Scottish malting cultivar characterized by round grains and a short, stiff culm [21], which protects the plants against lodging under typical nitrogenous fertilizer regimes.

## Material and Methods

### Plant material and growth conditions

The following barley (*Hordeum vulgare* L.) accessions were obtained from the Nordic Genetic Resource Center, Alnarp, Sweden (www.nordgen.org): near-isogenic line (NIL) BW042 (*ari-e*.*1*, NGB 20450), original mutant line *ari-e*.*1* (NGB 115846), BW043 (*ari-e*.GP, NGB20451), *ari-e*.*30* (NGB 115879), *ari-e*.*39* (NGB115889), *ari-e*.*119* (NGB 115931), *ari-e*.*156* (NGB 115966), *ari-e*.*166* (NGB115976), *ari-e*.*178* (NGB 115988), *ari-e*.*222* (NGB116031), *ari-e*.*228* (NGB 116038), and cv. Golden Promise (NGB23014). These lines were originally isolated as semi-dwarf lines after mutagenesis and are all non-transgenic. Further seed of cvs. Bowman, Foma, Bonus, and Maythorpe from Carlsberg Research Laboratory’s internal seed collection were used for this study.

For DNA isolation and sequence analyses of the barley *Dep1* (*HvDep1*) gene, plants were grown in the greenhouse at 16-h-light/8-h-dark cycles in Copenhagen (Denmark) and in Lund (Sweden). For allelic descriptions, plants were grown in the field near Middelfart, Denmark. The field studies did not require a special permit by national or local authorities, as all field trials were performed with non-GMO material that was grown according to local management practice on land either owned by the project partners or rented by them. The field studies did not involve endangered or protected species. Plants were sown on March 31^st^, 2014 and collected for phenotypic description of all alleles on July 24^th^, 2014.

### DNA isolation, PCR, and DNA sequencing

Leaf material (length approximately 1 cm) of 1-week-old seedlings was used for DNA isolation. DNA isolation and PCR reactions were performed using the REDExtract-N-Amp Plant Kit (Sigma-Aldrich, St. Louis, USA), following the manufacturer’s instructions. The following primer pairs were used for the amplification of the *HvDep1* gene:

IL307 (5’GCCTCCGCCTTCATTTCCAC’3) and IL275 (5’CCCTTCGCGGTTCGTGTTGG’3)

IL276 (5’CTACTGCTGCTGCTTCGTCG’3) and IL277 (5’CCGTCACCTGAGCACCTTCG’3)

IL278 (5’TGTTTCTCCGTGTGCCCTCC’3) and IL279 (5’GAGACTGGTTGAGCCCCTTC’3)

IL280 (5’CGTCAAGTCCAGTCCACACC’3) and IL281 (5’CGTGCGAGGATATTGGCGAC’3)

IL309 (5’GGCCATGCAAAGTTGTTGCC’3) and IL285 (5’TCCTCTGCTGCTGCCAGAAG’3)

IL286 (5’TGTCGTCGCTGTAAGGGTGC’3) and IL287 (5’CGGCGGAACAAGAGCCACAC’3)

TW001 (5’GCTGCTTCAAGATCCCTTCG’3) and TW002 (5’GCAGCAAGACAAAAGACAGC’3)

PCR conditions were: 94°C 2 min; 38 cycles of 94°C 45 s, 61°C 1 min, 72°C 1 min; 72°C 2 min.

The obtained PCR products were purified using the NucleoFast 96 PCR ultrafiltration system (Macherey & Nagel, Düren, Germany), and were sequenced at StarSEQ (Mainz, Germany).

### RNA-seq experiments

RNA-seq experiments were performed as described in S1 Table and in [22]

### The Derkado x B83-12/21/5 (DxB) population

A population of doubled haploids (DH) produced by anther culture of the F1 generation of a cross (B91-63) between the spring barley cv. Derkado and the breeding line B83-12/21/5 (DxB) [23–25] was used to study the agronomic effects of the *ari-e*.GP allele, which was carried by B83-12/21/5. The population had been grown in a series of trials from 1994–1997, as previously described [23, 26]. The population was also grown in additional agronomic trials in the Dundee area of Scotland in 1998, 1999, and 2002–2005 to form a matrix of 12 trials grown with fungicide protection and a malting barley nitrogen fertiliser application. The variables measured over all 12 trials considered for this study are listed in Table 1. A multiplication was conducted in 2007, when fresh DNA extractions were made for high throughput genotyping and grain from each plant sampled was retained as a reference stock.

**Table 1:** List of 11 agronomic, yield and yield related characters that were measured across 12 possible trial locations for the Derkado x B83-12/21/5 population.

Character	Eng96	Eng97	Scot94	Scot95	Scot96	Scot97	Scot98	Scot99	Scot02	Scot03	Scot04	Scot05
Head (days)	-	Y	Y	Y	Y	Y	-	Y	Y	Y	-	Y
Height (cm)	Y	Y	Y	Y	Y	Y	-	Y	Y	Y	Y	Y
Yield (t/ha)	Y	Y	Y	Y	Y	Y	Y	Y	-	Y	Y	Y
Glength (mm)	-	-	-	Y	-	Y	-	-	Y	Y	Y	Y
Gwidth (mm)	-	-	-	Y	-	Y	-	-	Y	Y	Y	Y
Width/Length (ratio)	-	—	-	-	-	-	-	-	-	-	-	-
TGW (g)	Y	-	Y	Y	Y	Y	-	-	Y	Y	Y	Y
Tillers/Plant (Nos)	-	-	Y	Y	Y	-	-	-	-	-	-	-
Grains/Ear (Nos)	-	-	Y	Y	Y	-	-	-	-	-	-	-
MSW (g)	-	-	Y	Y	Y	-	-	-	-	-	-	-
SPW (g)	-	-	Y	Y	Y	-	-	-	-	-	-	-

TGW = Thousand grain weight; MSW = weight of grain on the main stem (tallest); SPW = weight of grain produced by a single plant. Tillers/Plant, Grains/Ear, MSW, and SPW were all derived from the average of a grab sample of 5 plants taken from the middle of a yield trial plot. All the other characters were measured on yield trial plots or samples obtained from them after cleaning and grading. Y; data collected for a character for this specific year and location; -: no data collected for a trait for this specific year and location

Initial genotyping of the population with Simple Sequence Repeat (SSR), Amplified Fragment Length Polymorphism (AFLP) and related marker types has been described previously [23–25]. The entire population has since been genotyped with the 1536 SNP markers represented on the Barley Oligo Pooled Array 1 (BOPA1)[27] using the Illumina GoldenGate assay (Illumina Inc., San Diego, CA) and a sequence-derived marker to capture polymorphism in the *CslF6* gene. All the SNPs represented on BOPA1, together with the *CslF6* marker, are genic and the mapping described in this paper will therefore focus on the 508 markers that were polymorphic in the DxB population but with the inclusion of some key markers, such as *ari-e*.GP, *eph*, *mlo* and *sdw1*, already scored on the population because of their economic significance. A KASP marker was generated to genotypically score the population for the polymorphism in *HvDep1* and was added to the data set and a map covering all seven barley chromosomes generated using JoinMap 4 [28]. Seven of the BOPA1 SNP markers were unlinked with any other marker at LOD 2.0 but all the other markers grouped into seven linkage groups using the default parameters of JoinMap. Only one round of JoinMap was required to fit all the markers for five of the linkage groups, the exceptions being markers on chromosomes 1H and 5H where all three rounds were required but here only those that were fitted after the second round were considered (two markers were excluded).

### QTL mapping

A subset of 227 markers comprising BOPA1 SNPs and the key markers noted above was chosen on the basis that none was located within 1 cM of another marker. The phenotypic data used were the doubled haploid (DH) means for 142 individuals from each trial in which each variate was scored. The QTL mapping procedures in GENSTAT were used to detect genomic regions significantly associated with QTL main effects or QTL x E effects for each variate, with a genome wide 5% error threshold of 3.4 [29]. For the purposes of this paper, only the mapping results for chromosome 5H and the QTL detected whose confidence interval includes the *Ari-e* locus were considered.

### Allelic description of mutant and wild type lines

For determination of straw length, the longest tiller of each plant was selected and measured from the root neck to the collar of the ear. The length of each individual internode, the length of the ear (from collar to the tip of the uppermost grain) and the length of the awns (from the ear collar to the tip of the longest awn) as well as the number of grains were determined for the same tiller. In addition, the number of tillers and ears were counted per plant. Grains from each individual plant were threshed using a sieve of 2.2 mm width and weighed. Grain counting was done using a Data Count JR-D seed counter from Data Technologies (Jerusalem, Israel). The significance of observed phenotypic differences between mutant and wild type was tested by a two sided t-test for two independent populations with equal variance assumption in Microsoft Excel.

### Cloning of *HvDep1* variants and plant transformation studies

Full-length cDNA of the functional *HvDep1* gene (GenBank accession FJ039903.1) was synthesized and inserted into the *Eco*RV site of pUC57 by GeneScript (Piscataway, USA). The resulting plasmid was named pET-15b Dep1. The cDNA was PCR-amplified from pET-15b Dep1 (Dep1_cDNA_F1 5’-ATGGGGGAGGGCGCGGTGGT-3’; Dep1_cDNA_R1 5’-TCAACACAGGCACCCGCTAG-3’), purified and cloned into the Gateway compatible entry vector pCR8/GW/Topo (Invitrogen, Thermo Fisher Scientific, Waltham, MA USA) using the manufacturer’s instructions. The resulting plasmid was denoted pCR8_Dep1_cDNA. Using LR Clonase (Gateway® LR Clonase® Enzyme mix, Invitrogen, Thermo Fisher Scientific, Waltham, MA, USA), the cDNA was inserted from pCR8_Dep1_cDNA into the gateway destination vector pBRACT214 (pUBI::NOS; http://www.bract.org/constructs.htm) to generate the plant expression construct pEXPpBract214-Dep1_cDNA. To generate a truncated *HvDep1* cDNA, the full-length cDNA from pET-15b Dep1 was used as a template for PCR. Here the reverse primer included a TGA-translational-stop codon that would truncate the encoded protein by 143 amino acid residues from the COOH-terminus, leaving a protein of only 152 amino-acid residues (Dep1_cDNA_F1 5’-ATGGGGGAGGGCGCGGTGGT-3’; Dep1_cDNA_R2 5’-TCAGCAGCCGCAGCTCGGTTTG-3’). The PCR product was purified and inserted into pBRACT214 (pUBI::NOS) as described above to generate the plant expression construct pEXPpBract214-truncated_DEP1_cDNA.

For the transformation of barley, two *Agrobacterium tumefaciens* (AGL0) strains were generated. One strain carried the expression vectors pEXPpBract214-Dep1_cDNA and the helper plasmid pSOUP, while the other carried pEXPpBract214-truncated_DEP1_cDNA together with the helper plasmid pSOUP. Immature embryos of barley cv. Golden Promise were transformed in the plant transformation facilities at Flakkebjerg (Aarhus University, Denmark) according to the standard barley transformation protocol described by Holme et al. [30]. Transgenic plants were regenerated and analyzed in the plant transformation facilities at Flakkebjerg (Aarhus University, Denmark) using a hygromycin resistance selection screen in tissue culture as described in Holme et al. [30]. Additionally the presence of the transgene was confirmed by a standard PCR on genomic DNA using primers Hyg_F (5’-ACTCACCGCGACGTCTGTCG-3’) and Hyg_R (5’-GCGCGTCTGCTGCTCCATA-3’) which enable amplification of a fragment of the hygromycin resistance gene.

## Results

### Localization of the heterotrimeric G protein γ subunit genes in the barley genome

To identify heterotrimeric G protein γ-subunits in the barley genome, peptide sequences of the rice γ-subunits RGG1, RGG2, GS3 and DEP1 were used for blastp and tblastn searches of the barley genome database via the IPK barley Blast server (http://webblast.ipk-gatersleben.de/barley/), and of barley sequences deposited at NCBI GenBank. Identified barley sequences were used for a reverse blast search against the rice sequences at GenBank. Only when this search identified the original rice query sequence as its closest homolog, were both protein sequences considered to be orthologs. In addition to two canonical γ-subunits RGG1 and RGG2, an ortholog of the rice DEP1 sequence could be identified, while a barley ortholog of the GS3 subunit could not be found (Table 2). Thus, in contrast to rice, we conclude that the barley genome contains only one gene encoding an AGG3-type γ-subunit protein. This protein, ACI25445.1, was previously identified as *DEP1* homolog in barley [7, 8]. Further, the transcript abundance for the three barley γ-subunits was examined in the wild type cv. Morex, using data from an RNA-seq experiment that included 16 different tissues from various developmental stages [22], R. Waugh, unpublished[]. *HvDep1* was predominantly expressed in the rachis, the developing inflorescence, the lodicule, the palea, and the lemma. Expression of the γ-subunit 1 (*HvGg1*) was primarily detected in roots, the embryo and in developing grains, while expression of the γ-subunit 2 (*HvGg2*) was detected in all tissues included in the study (S1 Table).

**Table 2:** Barley orthologs of the rice heterotrimeric G protein γ-subunits.

Rice γ-subunit	NCBI accession	barley ortholog	IBSC [22] gene identifier (NCBI nucleotide accession #)	chromosomal position [31]
*RGG1* (γ-subunit 1)	BAD15277	BAJ90712.1	MLOC_81784 (AK359503)	5H; 120.35 cM
*RGG2* (γ-subunit 2)	ACY05516	BAJ98292.1	MLOC_80667 (AK367089)	not anchored
*GS3*	BAH89203	no ortholog identified		
*DEP1*	ACI25446	ACI25445.1	MLOC_52150	5H; 52.30 cM

**Fig 1:**
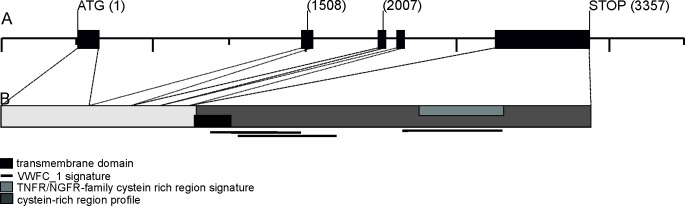
Structure of the *HvDep1* gene and the encoded polypeptide. (A) Graphic representation of the barley whole genome shotgun contig bowman_contig_881907 comprising the *HvDep1* coding sequence. 500-bp intervals are indicated with vertical bars. The coding sequence is shown as black boxes. The *HvDep1* coding sequence consists of five exons and four introns. The *ari-e* mutant lines cv. Golden Promise and BW043 (*ari-e*.GP) show an insertion of a single nucleotide in exon 2 after bp 1508, and lines *ari-e*.*39*, *ari-e*.*156* and *ari-e*.*166* show a nucleotide exchange in exon 3 at bp 2007 after the ATG start codon (details shown in Table 3). (B) Putative functional domains in the 295 amino-acid HvDEP1 protein. Using TMPRED (ch.embnet.org/software/TMPRED_form.html) and PROSITE (expasy.ch/prosite) a transmembrane domain, a putative tumor necrosis factor receptor (TNFR) ⁄ nerve growth factor receptor (NGFR) family cysteine-rich signature and von Willebrand factor type C (VWFC) cysteine-rich modules were predicted for HvDEP1. In a previous study [7] the Arabidopsis AGG3 γ-subunit was predicted to contain similar domains.

An integrated sequence-enriched genetic and physical map of the barley genome has been released [22] and is supplemented by a comprehensive set of barley genes, designated MLOCs, that are anchored to the physical map via whole genome shotgun (WGS) contigs of the barley cv. Morex. In this database, the *HvDep1* gene is listed as MLOC_52150, which only contains the coding sequence for the fifth exon. The full-length coding sequence was found to be covered by two separate Morex WGS contigs, morex_contig_38230 (exons 1, 2, 3 and 4) and morex_contig_37321 (comprising MLOC_52150). These are anchored to two closely linked locations in the draft assembly [31] (chromosome 5H at 50.44 and 52.30 cM, respectively). A full-length genomic sequence of *HvDep1* was identified on a single, unanchored WGS contig (bowman_contig_881907) of the barley cv. Bowman [22]. This full-length sequence was used for the graphic representation of the *HvDep1* gene structure shown in Fig 1.

### Identification of mutants at the *HvDep1* locus

To identify barley lines with mutations at the *HvDep1* locus, a near-isogenic line (NIL) collection of approximately 900 accessions representing the majority of the morphological and developmental variation described in barley [20] was screened. The recurrent parent used for creating this NIL collection was cv. Bowman, a North American two-row spring barley. The remaining introgressions of the original mutated genomes introduced into the Bowman genetic background can be linked to the barley physical map, because all lines of the NIL collection were genotyped with SNP markers [20]. This allows preliminary mapping of a mutant locus in the genome.

Rice loss-of-function mutants at the *DEP1* locus are known to be of semi-dwarf stature, including dense ears and erect panicles with an increased number of branches [8]. Barley *HvDep1* mutants were expected to have a similar semi-dwarf phenotype, in spite of obvious differences in the architecture of the inflorescences in rice and barley. On the barley physical map, *HvDep1* is located on chromosome 5H at approximately 50 cM. Thus, the Bowman NIL collection was screened for lines with a semi-dwarf stature and mutant donor introgressions that harbor an interval on chromosome 5H, including the region around 50 cM. One accession that matched both criteria was line BW043 (*ari-e*.GP), which contained an introgression on chromosome 5H that spanned the region between 28.3 and 92.2 cM originating from the Scottish malting-barley cv. Golden Promise (S2 Table). Sequencing of the *HvDep1* gene in BW043 (*ari-e*.GP) and Golden Promise revealed a 1-bp (adenine) insertion after position 1508 in the second exon of the *HvDep1* gene (Fig 1A). The insertion translates into a truncated polypeptide consisting of 54 native amino-acid residues followed by 12 residues that are different from the sequence of the wild type protein. The 1-bp insertion was found neither in the barley cv. Maythorpe, from which Golden Promise was originally derived through mutagenesis, nor in cv. Bowman, the recurrent parent of the NIL BW043 (*ari-e*.GP). A recent genotyping-by-sequencing (GBS) study on a recombinant inbred-line population derived from a cross between the barley cvs. Golden Promise (*ari-e*.GP) and the six-rowed Morex (*Ari-e*) mapped the *ari-e*.*GP* mutation at a position around 52 cM on chromosome 5H [32]. This position coincides with the anchored position of MLOC_52150, the gene model for *HvDep1*, at 52.30 cM on chromosome 5H (Table 2), and suggested *HvDep1* was a strong candidate for *Ari-e*.GP.

A set of allelic *ari-e* mutant lines deposited in barley mutant gene bank collections was used (Table 3) to obtain further proof that *HvDep1* was *Ari-e* in barley. In total, 10 different *ari-e* alleles are known, of which two alleles are also available as Bowman NILs. The *HvDep1* gene, including all five exons and the four introns, was amplified by PCR from genomic DNA and sequenced in all lines, together with the respective wild type cvs. Bonus, Foma, and Bowman (Table 3). The *HvDep1* gene sequences of all parental lines were found to encode a full-length protein identical to that of accession ACI25445.1. In contrast to the parental lines, a cytosine to thymine change in exon 3 in *ari-e* mutant lines *ari-e*.*39*, *ari-e*.*156*, and *ari-e*.*166* causes a premature stop codon and thereby an immediate truncation of the derived polypeptide after amino-acid residue no. 72. In lines *ari-e*.*1*, *ari-e*.*30*, and *ari-e*.*119*, attempts to amplify seven different fragments of the *HvDep1* gene were unsuccessful. Similarly, in line BW042, the Bowman NIL of the original mutant *ari-e*.*1*, it also proved impossible to amplify *HvDep1*, suggesting that is the gene is deleted in *ari-e*.*1*, *ari-e*.*30*, and *ari-e*.*119*. The mutations in *ari-e*.*1* and *ari-e*.*119* were radiation induced [33], a process that is known for predominantly inducing deletions.

**Table 3:** Description *of breviaristatum-e* (*ari-e*) mutant lines.

barley line/mutant allele	mother cultivar	mutation	effect at protein level	phenotype during vegetative growth
BW042/*ari-e*.*1*	Bowman (NIL)	gene deleted	no protein	erect
*ari-e*.*1*	Bonus	gene deleted	no protein	erect
BW043/*ari-e*.GP	Bowman (NIL)	insertion of A after position 162 in the cDNA	54 native residues followed by TSLTFCLLERGK	erect
Golden Promise/*ari-e*.GP	Maythorpe	insertion of A after position 162 in the cDNA	54 native residues followed by TSLTFCLLERGK	erect
*ari-e*.*30*	Bonus	gene deleted	no protein	erect
*ari-e*.*39*	Bonus	C to T nonsense mutation at position 217 in the cDNA	truncated protein consisting of 72 native amino-acid residues	erect
*ari-e*.*119*	Foma	gene deleted	no protein	erect
*ari-e*.*156*	Foma	C to T nonsense mutation at position 217 in the cDNA	truncated protein consisting of 72 native amino-acid residues	erect
*ari-e*.*166*	Foma	C to T non-ense mutation at position 217 in the cDNA	truncated protein consisting of 72 native amino-acid residues	erect
*ari-e*.*178*	Foma	no mutation found in *HvDep1*	none	wild type
*ari-e*.*222*	Foma	no mutation found in *HvDep1*	none	wild type
*ari-e*.*228*	Foma	no mutation found in *HvDep1*	none	wild type

**Fig 2:**
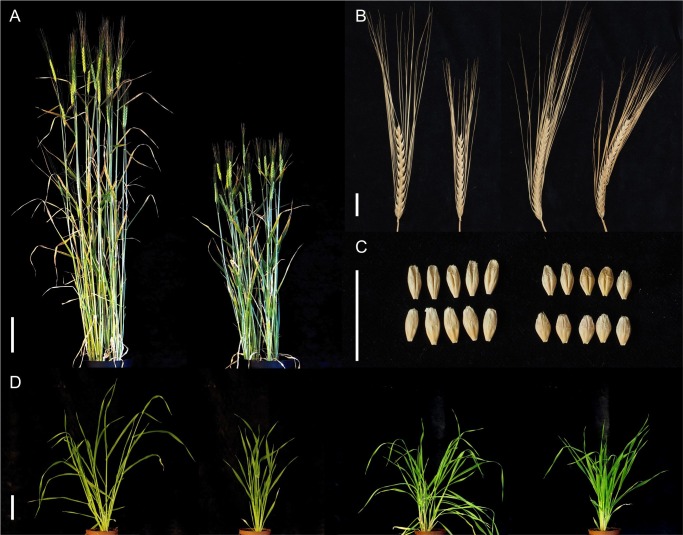
Phenotype of barley *breviaristatum-e* (*ari-e*). (A) Comparison of overall plant height between the wild type cv. Bowman (left) and *ari-e*.*1* mutant NIL BW042 (right); scale bar: 10 cm (B) Spikes of barley lines Bowman, BW042 (*ari-e*.*1*), Maythorpe, Golden Promise (from left to right). Mutant lines are characterized by reduced awn length. (C) Seed shape of cvs. Maythorpe (left) and Golden Promise (right). (D) Gross morphology in the vegetative phase (from left to right: Bowman, BW042 (*ari-e*.*1*), Maythorpe, Golden Promise). Mutant lines appear more upright due to narrow leaf angles; scale bar: 10 cm.

All the mutations identified in the *HvDep1* gene of the different *ari-e* mutant lines represent loss-of-function alleles. The coding sequence is either completely deleted or stop codons are introduced in its proximal region. The translated truncated polypeptides lack the γ-domain, the putative transmembrane domain and the COOH-terminal cysteine-rich domain, making interaction with the β-domain unlikely. Thus, a functional HvDEP1 protein is most likely not present in the mutant lines. Fig 2 summarizes the impact of the *Hvdep1* loss-of-function mutations on barley plant architecture. Plants are characterized by reduced height (Fig 2A) as well as shorter awns (Fig 2B). The grains of *ari-e* mutant plants are smaller than those of the corresponding wild type cultivars (Fig 2C). In addition, the mutants are characterized by narrowed leaf angles that lead to an upright or erect, compact phenotype during the vegetative growth period (Fig 2D), a Mendelian characteristic that was originally used to map *ari-e*.*GP* to chromosome 5H [34].

**Fig 3:**
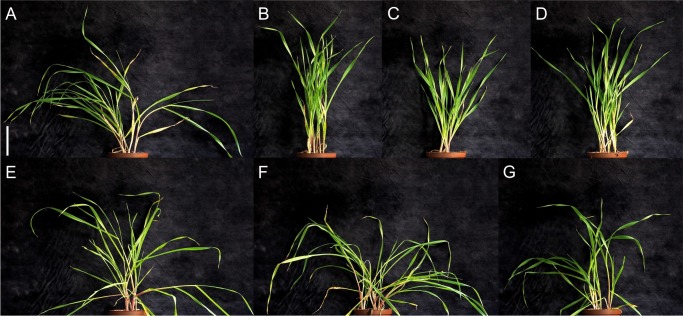
Phenotype of *ari-e* mutant lines in a cv. Foma genetic background. Barley lines Foma (A), *ari-e*.*119* (B), *ari-e*.*156* (C), *ari-e*.*166* (D), *ari-e*.*178* (E), *ari-e*.*222* (F), *ari-e*.*228* (G) during the vegetative growth phase when grown in the greenhouse. All pictures show three plants in one pot sown on the same day. Only *ari-e*.*119*, *ari-e*.*156* and *ari-e*.*166* show the characteristic erect phenotype of *ari-e* mutants and mutations in *HvDep1* were only found in these lines. Scale bar: 10 cm

It is notable that no changes were identified in the nucleotide sequence of the *HvDep1* genomic sequence of mutant lines *ari-e*.*178*, *ari-e*.*222*, and *ari-e228*. Therefore, the phenotype of those three lines was compared to that of lines *ari-e*.*119*, *ari-e*.*156*, and *ari-e*.*166*, as all six mutant lines were generated in the same genetic background, namely cv. Foma [35]. Only the mutant lines *ari-e*.*119*, *ari-e*.*156*, and *ari-e*.*166*, and not lines *ari-e*.*178*, *ari-e*.*222*, and *ari-e*.*228*, had the characteristic upright and erect growth habit of *ari-e* mutants (Fig 3), indicating that the latter lines do not represent *ari-e* mutants but instead carry an unknown mutation in a gene that causes a semi-dwarf phenotype but not the characteristic erect growth habit of *ari-e* mutants.

### The *Ari-e* locus controls plant height, grain size and yield

To understand how *Ari-e* controls plant height and yield in barley, a population derived from a cross between the spring barley cv. Derkado (*Ari-e*.GP) and the breeding line B83-12/21/5 (DxB), which carries the *ari-e*.GP allele [23], was used. The morphological scoring (S3 Table) of the *ari-e*.GP phenotype in the DxB population resulted in the *Ari-e* locus being mapped at 25.8 cM between the BOPA1 SNPs 11_20265 and 11_20392, which are located at position 48.38 in the barley draft assembly [31], on chromosome 5H. The 1-bp insertion identified in the *HvDep1* sequence in cv. Golden Promise co-segregated with the *ari-e*.GP morphological score, confirming that this polymorphism conferred the phenotype exemplified by cv. Golden Promise.

A highly significant QTL for plant height, TGW, grain length, grain width to length ratio, grain yield, and heading date was detected with a peak coincident with the *Ari-e* locus. The *ari-e*.*GP* allele reduced height by between 6.2 and 9.2 cm over all 11 environments tested (Table 1). The height QTL was the most significant association with the *Ari-e* locus. But very highly significant effects for TGW, grain length and the grain width to length ratio were also detected. The effect of the mutant *Hvdep1* allele was to reduce TGW and grain length with the reduction in the latter resulting in an increase in the width to length ratio, i.e. more rounded grain. These effects were significant in each environment in which the characters were measured, with the mutant *Hvdep1* allele reducing TGW by between 4 and 1.7 g across 9 environments.

**Fig 4:**
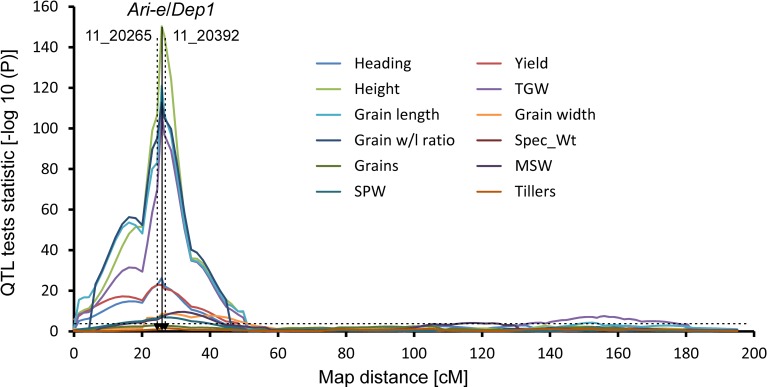
QTL profiles for 12 agronomic and yield related characters for chromosome 5H from the Derkado x B83-12/21/5 map. QTL profiles for 12 agronomic and yield related characters for chromosome 5H from the Derkado x B83-12/21/5 map. The position of the *Ari-e*/*Dep1* locus is indicated by the solid vertical arrow and the flanking SNP markers by vertical dashed arrows. BOPA1 SNPs 11_20265 and 11_20392 are placed at position 48.38 cM in the barley draft assembly [31]. The significance threshold (3.4) for a QTL in this population is indicated by the straight dashed horizontal line. SPW: Single Plant Weight of grain; TGW: Thousand Grain Weight; Spec_WT: specific grain weight [kg/hl]; MSW: Main Stem Weight, weight of grain from the tallest tiller of plants.

Further, a grain width QTL was detected with a peak at 28.5cM. Its confidence interval overlaps with the location of *HvDep1* but it is a cross-over interaction with the effect of the *ari-e*.*GP* allele being positive in one environment, negative in three and non-significant in two. The effect of the *ari-e*.*GP* allele at this grain width QTL is weak ranging from -0.045 to +0.028mm and its peak–log10(P) score is also low (Fig 4A) whereas the effect of the *ari-e*.*GP* allele at the grain length QTL is much stronger. The net result is that the *ari-e*.*GP* allele at the grain width to length ratio is also strong, as it increases the ratio in all 6 environments in which the parameter was tested by between 0.018 and 0.026. However, no significant associations of the mutant allele with grain number per spike or tillers (side branches) per plant could be detected, suggesting an absence of any compensatory effect for the reduced TGW. In addition, the mutant *Hvdep1* allele significantly reduced heading date in nine of the ten environments in which it was measured by an average of 1.5 days. The peak of the QTL for grain yield on chromosome 5H is just distal to the *Ari-e* locus, which is within the confidence interval for the QTL (Fig 4). Accordingly, it can be concluded that the locus has a significant effect upon grain yield per ha. The effect of the *ari-e*.GP allele on grain yield is, however, variable insofar as it significantly increased or decreased yield at three and four sites, respectively, out of the 11 in which the character was measured. The size of the effect of the mutant allele ranged from -0.20 to +0.23 t/ha. The mutant *ari-e*.GP allele significantly shortened the time between sowing and heading, a proxy that is used for the determination of flowering time, by up to 1.5 days in 9 of the 10 environments in which the character was measured.

### Mutations at the *HvDep1* locus have pleiotropic effects on plant architecture

Given that the *dep1* mutation in rice confers improved yield through an increased number of grains per plant [8], possible effects of the loss-of-function mutations at the *Ari-e* locus on grain number per plant were investigated. To this end, a field experiment in Denmark was performed during spring/summer 2014, which included all known *ari-e* loss-of-function mutants (Table 4). First morphological features characteristic for the *ari-e* mutant phenotype such as straw and awn length were measured. As expected it was found that all *ari-e* alleles significantly reduced straw length in each genetic background considered. The same is true for the awn length, which was significantly reduced in all *ari-e* mutant lines. The extent of the decrease in straw and awn length varied between genotypes. As the awn length is dependent on the length of an individual spike, the awn length was normalized to the spike length. This was achieved by calculating the ratio of the combined length of awn and spike and the length of the spike only (Table 4).

**Table 4:** Phenotype of field grown *ari-e* mutant lines in comparison to the respective mother cultivar.

	Bowman	BW042 (*ari-e*.*1*)	BW043 (*ari-e*.GP)	May-thorpe	Golden Promise	Foma	*ari-e*.119*	*ari-e*.156*	*ari-e*.166*	Bonus	*ari-e*.1*	*ari-e*.30*	*ari-e*.39*
Number of plants measured	19	17	16	13	15	13	10	11	17	19	15	18	19
straw length [cm]	94.3 ± 5.1	67.7 ± 5.5	67.2 ± 2.6	86.8 ± 3.4	67.8 ± 3.5	93.2 ± 3.6	64.1 ± 2.9	79.7 ± 3.6	78.1 ± 6.1	93.6 ± 3.8	74.1 ± 1.3	68.2 ± 4.5	78.7 ± 5.7
significance^#^		< 0.001	< 0.001		< 0.001		< 0.001	< 0.001	< 0.001		< 0.001	< 0.001	< 0.001
spike length [cm]	8.7 ± 0.6	7.3 ± 0.5	7.1 ± 0.8	9.3 ± 0.5	9.0 ± 0.8	9.5 ± 0.9	9.2 ± 1.2	9.5 ± 1.1	8.5 ± 1.4	10.8 ± 1.0	10.2 ± 1.0	9.0 ± 0.9	9.5 ± 1.1
significance^#^		< 0.001	< 0.001		0.215		0.411	0.979	0.034		0.074	< 0.001	< 0.001
ratio length (spike + awn)/spike	2.39 ± 0.12	1.96 ± 0.09	1.89 ± 0.26	2.20 ± 0.10	1.70 ± 0.13	2.26 ± 0.15	1.80 ± 0.30	1.69 ± 0.10	1.75 ± 0.11	1.96 ± 0.10	1.52 ± 0.07	1.74 ± 0.09	1.65 ± 0.09
significance^#^		< 0.001	< 0.001		< 0.001		< 0.001	< 0.001	< 0.001		< 0.001	< 0.001	< 0.001
Number of grains per spike	21.26 ± 1.24	19.47 ± 1.55	18.94 ± 1.77	29.00 ± 1.96	29.13 ± 2.13	29.23 ± 1.42	30.70 ± 1.34	33.18 ± 2.89	27.19 ± 2.61	30.68 ± 1.57	29.07 ± 2.12	30.33 ± 2.20	30.74 ± 2.10
significance^#^		< 0.001	< 0.001		0.865		0.020	< 0.001	0.018		0.015	0.578	0.931
thousand grain weight (TGW) [g]	48.9 ± 3.1	40.2 ± 2.0	38.3 ± 3.0	50.78 ± 3.39	41.54 ± 4.74	47.94 ± 3.94	35.58 ± 2.06	35.04 ± 7.55	33.84 ± 4.04	48.15 ± 2.80	41.06 ± 1.41	35.19 ± 2.40	34.74 ± 3.02
significance^#^		< 0.001	< 0.001		< 0.001		< 0.001	< 0.001	< 0.001		< 0.001	< 0.001	< 0.001

*Mutants *ari-e*.*119*, *ari-e*.*156*, and *ari-e*.*166* are derived from Foma whereas *ari-e*.*1*, *ari-e*.*30* and *ari-e*.*39* were induced in Bonus

^#^p values returned from t test between a mutant line and the respective mother cultivar (compare Table 3), a value of p < 0.05 was considered significant

**Fig 5:**
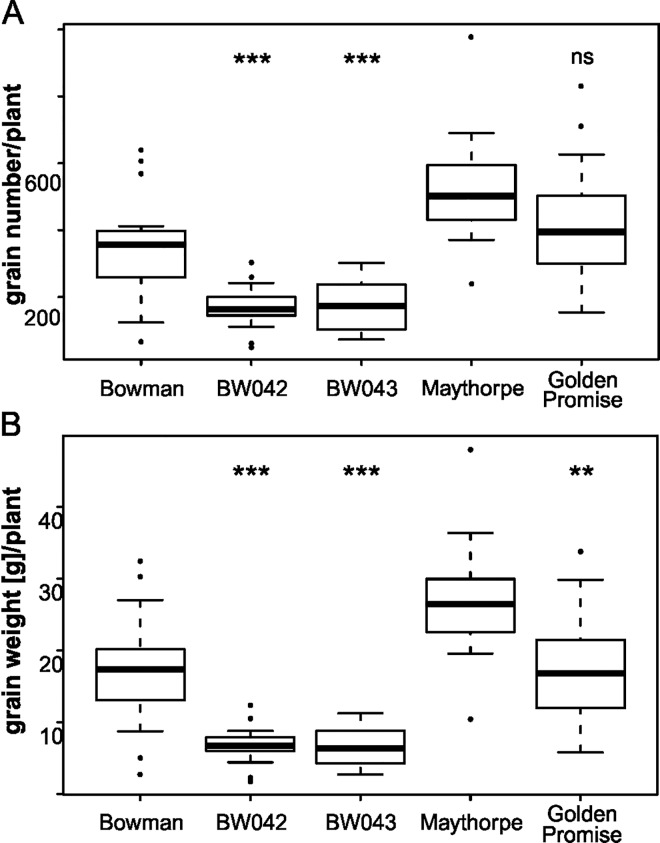
Grain yield measured in field trials in Denmark. Values obtained for (A) grain number per plant and (B) grain weight per plant from field grown lines (summer 2014 in Denmark). The number of individuals considered for each line is given in Table 4. T-tests were performed to test for significant differences between respective wild type and mutant lines. Statistically significant differences are indicated by asterisks within the figure (threshold: * p < 0.05, ** p < 0.01, *** p < 0.001). Average values concerning grain number per plant and grain weight per plant respectively were 49.4% and 40.3% for BW042 and 51.7% and 39.7% for BW043 in comparison to Bowman and 81.6% and 66.7% for Golden Promise in comparison to Maythorpe.

Further, both the number of grains per plant and the total grain weight per plant were investigated (Fig 5). For BW042 (*ari-e*.*1*) and BW043 (*ari-e*.*GP*), the average grain number per plant was only about 50% of that of Bowman, and the grain weight per plant decreased by 60% (Fig 5). This adverse effect on grain yield per plant was less pronounced in the genetic background of cv. Maythorpe. Nevertheless, the grain weight per plant for cv. Golden Promise was only 67% of the value observed for cv. Maythorpe. The different *ari-e* mutants displayed a wide variation in spike length and number of seeds per spike (Table 4), which may explain some of the variation seen for the grain number of plants. In the case of cv. Golden Promise, no difference regarding spike length could be observed in comparison to cv. Maythorpe and the number of grains per spike was not affected. In contrast, the NIL BW043 (*ari-e*.*GP*) had shorter spikes and fewer grains per spike compared with cv. Bowman (Table 4), which was unexpected because BW043 carries the same allele, *ari-e*.*GP*, as cv. Golden Promise. In the *ari-e* mutant lines derived from Foma and Bonus the data shows a trend for a slight reduction in spike length, while the number of grains per spike remained unaffected. This might be due to a reduction in rachis internode length in these lines. Further, the TGW was lower in all mutant lines.

### Complementation of *ari-e* in cv. golden promise with full length and truncated *HvDep1* variants

**Fig 6:**
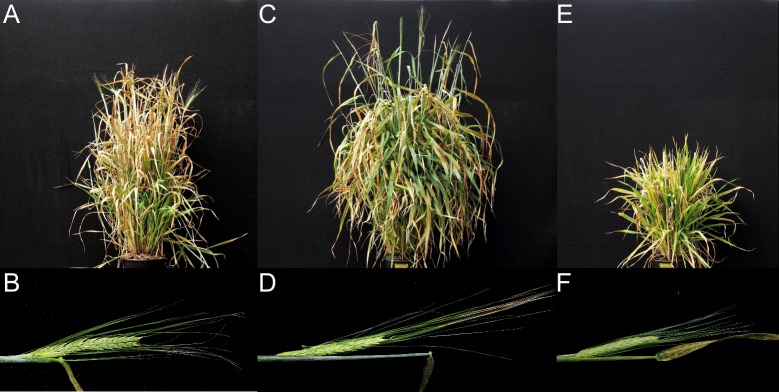
Complementation of *ari-e* in Golden Promise with full length and truncated forms of *HvDep1*. (A) and (B) Phenotype of Golden Promise, carrying the *ari-e*.GP loss-of-function allele, compared to (C) and (D) Golden Promise full-length pUBI:*HvDep1* transformants and (E) and (F) Golden Promise pUBI: *HvDep1*Δ152 transformants. In the later, only the first 152 amino-acid residues of HvDEP1 are translated. Plants with the pUBI: *HvDep1*Δ152 transformation developed very few spikes and no fertile seeds.

All mutant lines carrying *ari-e* alleles with an *Hvdep1* loss-of-function genotype, like that of cv. Golden Promise, are characterized by a short culm. In rice however, *DEP1* alleles encode truncated proteins that most probably retain some functional features that have been selected by plant breeders due to their beneficial influence on grain yield [8]. A transgenic approach was used to clarify whether truncations of different length of the HvDEP1 protein would confer a different effect on the barley phenotype (compare S1 Fig). Firstly, the loss-of-function mutant Golden Promise (*Hvdep1*), the standard cultivar for transgenic studies in barley [36] was transformed with a functional *HvDep1* allele that should reconstitute the wild type phenotype. For this experiment, the wild type *HvDep1* allele from cv. Maythorpe was inserted into a plant transformation vector under the control of an ubiquitin promoter for constitutive expression in all plant tissues. Transgenic plants were selected on the basis that they were resistant to the antibiotic hygromycin, with the resistance conferring gene linked to the transgene in the expression vector, as well as the presence of a PCR amplicon of the transgene in mature leaves. In total, six independent transgenic plants carrying the full-length *HvDep1* construct were regenerated. Four of these plants showed a reconstituted cv. Maythorpe-like phenotype, which is characterized by taller and less erect plants with long awns (Fig 6). This indicated that ectopic overexpression of *HvDep1* reconstituted the predominant traits of cv. Maythorpe. The cv. Maythorpe-like phenotype was also observed in the T1 generation of these plants. Two T_0_ transgenic plants had the phenotypic characteristics of cv. Golden Promise, possibly because the *HvDep1* expression construct was not transcribed properly in these plants.

Additionally cv. Golden Promise was transformed with a truncated Δ*Hvdep1* allele under the control of a ubiquitin promoter, which translates into a truncated protein of the first 152 amino-acid residues of the *HvDep1* gene product and is equivalent to the allele found in high yielding rice cultivars [8]. Fourteen regenerated T_0_ transgenic plants had a severe dwarf phenotype (Fig 6). Introduction of the Δ*Hvdep1* expression vector under control of the ubiquitin promoter into cv. Golden Promise had a drastic impact on the overall appearance of the plants, including repression of culm elongation, reduced formation of flag leafs and deficient development of fertile spikes. This severe phenotype is different from that observed in the barley *ari-e* mutant cv. Golden Promise (Fig 6B) and suggests that the truncated version of the protein in barley might retain activity and has a different function than the full-length protein.

## Discussion

### The loss-of-function mutation at the *Ari-e* locus in barley resulted in a successful malting cultivar

The malting barley cv. Golden Promise carries the *ari-e*.*GP* mutation at the *Ari-e* semi-dwarfing locus. The plant was isolated after γ-ray mutagenesis of the cv. Maythorpe in 1956 and it is an important induced mutant, developed with the aim of introducing a short, stiff culm into a malting barley background [21, 37].

Its semi-dwarfism coupled with early heading, made cv. Golden Promise particularly suitable for agriculture in Northern Britain where it was widely adopted. The current study shows that the semi-dwarf stature of Golden Promise is due to a loss-of-function mutation in the *HvDep1* gene, the barley ortholog of rice *Dep1* [8]. The *Ari-e* semi-dwarfing locus was identified in attempts to find *HvDep1* mutants in a collection of barley NIL mutant lines by combining phenotypic information and data on the genomic location of mutant loci, as previously demonstrated by Dockter et al. [38]. Evidence that the *HvDep1* gene is identical to *Ari-e* came from the identification of severe mutations in the *HvDep1* gene in a series of allelic *ari-e* mutants and through transgenic complementation studies.

Complementation of a mutated gene with its wild type allele provides direct evidence that the mutant phenotype is conferred by mutations in the studied gene. This critical link is important when studying induced mutants like cv. Golden Promise, primarily because the mutagenic treatment induces thousands of random mutations across the genome. Complementation has rarely been performed in barley, mainly due to limitations regarding transformable cultivars. In fact, cv. Golden Promise remains the cultivar routinely used for high-throughput barley transformation studies [39, 40]. Therefore it was hypothesized that the mutation causing the semi-dwarf phenotype of cv. Golden Promise might confer the superior transformability on this cultivar [41]. However, transformation studies with cv. Maythorpe, the mother cultivar of cv. Golden Promise, revealed similar transformability [41], thus excluding the *Ari-e* locus and thereby the *HvDep1* gene as the critical factor conferring increased transformability on cv. Golden Promise.

### *HvDep1* is a positive regulator of culm and seed elongation in barley and influences grain yield

Based on the finding that the grains of barley *ari-e* loss-of-function mutants are shorter combined with the plant’s semi-dwarf stature, we conclude that *HvDep1* is a positive regulator of culm and seed elongation, similar to the Arabidopsis *AGG3* gene, that functions as a positive regulator of organ growth [7]. The present study reveals further that the phenotype caused by a loss-of-function mutation of *HvDep1* is partly dependent on the plant’s genetic background. While all *ari-e* mutants shared some phenotypic characteristics, e.g. reduced culm and awn length and a lower TGW, other features differed between genotypes. The *ari-e*.GP allele did not confer fewer grains per spike in cv. Golden Promise compared to cv. Maythorpe but did in the NIL BW043 (*ari-e*.GP) compared to cv. Bowman. In addition, the number of grains per plant was significantly reduced in the *ari-e* mutant lines with a cv. Bowman genetic background, while no such reduction was observed between cvs. Golden Promise and Maythorpe. Cv. Bowman carries the *eam6* allele that leads to early maturity [42] and has a significantly earlier heading date than cv. Golden Promise under long day growth conditions. It is tempting to speculate that the concomitant presence of the two earliness inducing alleles *eam6* and *ari-e*.GP in BW043 leads to a reduction of the number of grain per ear and hence per plant as observed in this study. Still, as the *ari-e*.*GP* reduces the time to heading in Golden Promise only by 1.5 days it remains elusive if this is sufficient to induce the observed effects.

QTL mapping indicates that the effect on yield of the *ari-e*.GP mutation is highly dependent on the growth environment. In some years the mutation positively influences yield, in others it is associated with a reduction. Underlying these differences could be the earlier maturity of plants carrying the *ari-e*.GP allele, as observed in most locations, to reduce local late-season water stress in the three environments where the allele was associated with higher yield.

Whilst heading date is often measured in academic experiments, it is crop maturity that is more critical in maximizing yield potential and Golden Promise was always noted as an early maturing cultivar. For instance, it was two days earlier maturing than any other cultivar on the 1979 Scottish Agricultural Colleges Recommended List and 8 days earlier than Maris Mink, which carried the semi-dwarf allele at the *sdw1* locus. (Anon., 1979). In a series of European Trials grown in 2009 and 2010, cv. Golden Promise headed approximately 3 days earlier than cv. Optic but matured almost 5 days earlier (Flavell et al., unpublished data). Clearly, the early maturity associated with the *Hvdep1* mutant allele would be advantageous under a terminal drought situation and that coupled with the resistance to lodging appears to be the reason behind the yield advantage also associated with the mutant allele in the trials Eng96, Eng97, and Scot97 (compare Table 1). Inspection of the climate anomalies charts available at http://www.metoffice.gov.uk/public/weather/climate-anomalies/#?tab=climateAnomalies shows either an increased mean temperature in June and or July or a dramatic increase in rainfall in these critical months for grain filling. Generally, the temperature and rainfall patterns in the UK are such that late season drought does not occur nor is there so much rainfall as to induce widespread lodging but, when either occurs, there is an advantage to having the early maturity associated with the mutant *Hvdep1* allele. Under more normal conditions it is more advantageous to maximize the grain-filling period to achieve high yield, hence under these conditions the mutant allele would have a negative effect on yield.

The effect of genetic background and growth conditions on the impact of *HvDep1* on grain yield is noteworthy, especially in the context of the ongoing discussion on how the *Dep1* locus operates in rice. In the latter, several mutant alleles at the *DEP1* locus encode COOH-terminally truncated DEP1 proteins: *dep1* [8], *qpe9-1* [18], the identical alleles Dn1-1 and Dn1-2 [43], and Dn1-3, where the COOH-terminus of the deduced protein sequence is replaced by an alternative sequence due to a frame shift mutation [43]. While an increase in grain number per plant was observed in *dep1* rice mutant lines due to an increase in panicle branching [8] the *qpe9-1* allele did not confer increased branching and was associated with a reduced number of grains per plant [18, 44].

These contrasting findings are unlikely due to different functions of the proteins encoded by the mutant alleles, as the truncations caused by the *dep1* and *qpe9-1* mutations at the protein level confer only one amino-acid residue difference in length. Although an explanation for these discrepancies remains elusive, it is notable that the mutations were studied in different genetic backgrounds. Another study focused on the effect of the *Dn1-1* allele on panicle branching in two different rice cultivars [28]. In cv. Nipponbare, the number of primary branches remained unaltered, but it increased in Koshihikari [43]. Accordingly, several observations suggest that, like in barley, mutations at the *DEP1* locus in rice result in different phenotypes depending on the genetic background.

Despite the obvious similarities between *dep1* mutants in both crops, it should be noted that there are major differences between the rice and barley mutations in *Dep1*. In all *ari-e* mutant barley plants the *HvDep1* gene is either completely deleted or contains translational stop codons that confer truncations in the encoded protein such that deletions occur not only in the cysteine rich repeat, as seen in rice, but also in the putative transmembrane domain and parts of the γ-domain. Accordingly, it is highly unlikely that *ari-e* mutants possess DEP1-type γ-subunits with the capacity to interact with the β-subunit of the heterotrimeric G-protein. This suggests that the barley *ari-e* mutations represent loss-of-function mutations. It should, however, be noted, that all barley mutant lines described in this study were isolated after induced mutagenesis. Therefore, it remains possible that natural mutation events conferring gain-of-function mutations as described in rice, have occurred but are yet undiscovered.

In the described rice mutants, however, larger regions of the encoded protein remain intact and hence the proteins might be partly functional. This is supported by the observation that rice *dep1* is a gain-of-function mutation [8], whereas the very similar *qpe9-1* allele represents a loss-of-function mutation [18]. Due to these contrasting findings, it is unclear what kind of effect could be expected from the expression of a similar truncated protein in barley. For that reason the barley cv. Golden Promise, which carries the *ari-e*.*GP* loss-of-function allele, was transformed with an expression construct directing synthesis of a truncated protein of 152 amino-acid residues. The resulting transformants had a severe dwarf phenotype, indicating that the truncated version of the encoded protein was indeed active *in planta*. The transformants had a phenotype that was remarkably different from both the cv. Golden Promise (no HvDEP1 protein) and the cv. Maythorpe (full-length HvDEP1 protein) plant architecture, and it is hence conceivable that barley mutations resulting in a truncated protein represent gain-of-function mutations. Still, it cannot be excluded that the observed effect is a result of the ectopic overexpression under a constitutive promoter. The RNA-seq data revealed that the expression profile of the three γ-subunits is specific for developmental stages and tissues. Overexpression of the Δ*HvDep1* gene might hence impair the formation of functional complexes with either HvGG1 or HvGG2 and the β-subunit of the signaling complexes especially in tissues where the endogenous *HvDep1* gene is not expressed in wild type plants.

It is worth mentioning the hypothesis that barley and wheat have evolved with a gain-of-function variant of the DEP1 protein similar to that of the rice *dep1* allele [8], given that the DEP1 proteins of wheat (295 amino-acid residues), its diploid wild progenitor *Triticum urartu* (283 residues) and barley (295 residues) are notably shorter than the rice DEP1 protein (426 residues). This possibility is supported by an observation in wheat, where RNAi-mediated down-regulation of *TaDep1* resulted in longer and less compact spikes [8], which contrasts the observations made for barley described here. Nevertheless, as the deletion found in *dep1* rice mutants is a COOH-terminal truncation while the differences in length in wheat and barley compared to rice rather result from internal deletions [8], they are most likely not functionally equivalent

## Supporting Information

S1 Table RNA-seq expression data of the heterotrimeric G protein γ-subunits of barley. (XLSX)

S2 Table Localization of the introgression region present in Bowman NILs BW042 and BW043 on the barley physical map. (XLSX)

S3 Table Derkado x B83-12/21/5 population morphological scores. (XLSX)

S1 Fig PCR to confirm the presence of the hygromycin resistance gene in transgenic barley lines transformed with the full length or truncated *HvDep1* gene. Agarose gel to visualize results of a PCR from genomic DNA to confirm the presence of the hygromycin resistance gene in wild type and transgenic barley lines. The gel was loaded with PCR reactions from different barley lines as follows: lane 1; Golden Promise (untransformed control); lanes 2–3: lines transformed with full-length *HvDep1*; lanes 4–6: lines transformed with truncated Δ*Hvdep1*; lanes 7–11: lines transformed with full-length *HvDep1*.
(TIF)
