# Supplementary figures and images for: HvDep1 Is a Positive Regulator of Culm Elongation and Grain Size in Barley and Impacts Yield in an Environment-Dependent Manner
Source: PLoS One. 2016 Dec 22;11(12):e0168924. doi: 10.1371/journal.pone.0168924 (PMC5179111; doi:10.1371/journal.pone.0168924)

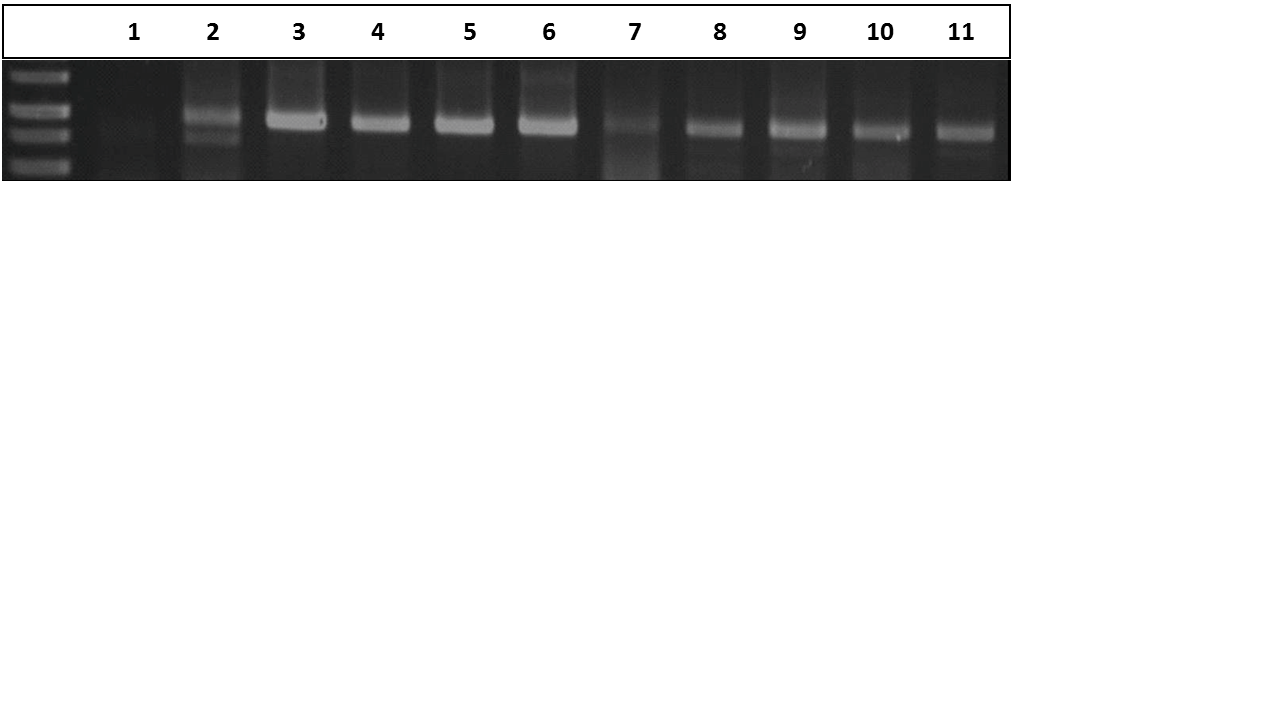

Supplement: S1 Fig — Agarose gel to visualize results of a PCR from genomic DNA to confirm the presence of the hygromycin resistance gene in wild type and transgenic barley lines. The gel was loaded with PCR reactions from different barley lines as follows: lane 1; Golden Promise (untransformed control); lanes 2–3: lines transformed with full-length HvDep1; lanes 4–6: lines transformed with truncated ΔHvdep1; lanes 7–11: lines transformed with full-length HvDep1. (TIF) [file pone.0168924.s004.tif]
